# Supplementary material for: Cellular Imaging and Time-Domain FLIM Studies of Meso-Tetraphenylporphine Disulfonate as a Photosensitising Agent in 2D and 3D Models
Source: Int J Mol Sci. 2024 Apr 11;25(8):4222. doi: 10.3390/ijms25084222 (PMC11050357; doi:10.3390/ijms25084222)
Supplement: Supplementary file 1 [file ijms-25-04222-s001.zip › ijms-2924650-supplementary.pdf]

International Journal of
Molecular Sciences

MDPI

Supplementary

Cellular Imaging and Time-Domain FLIM Studies of Meso-Tetraphenylporphine Disulfonate as a Photosensitising Agent in 2D and 3D Models

Andrea Balukova [1], Kalliopi Bokea [1], Paul R. Barber [2,3], Simon M. Ameer-Beg [3], Alexander J. MacRobert [1,*] and Elnaz Yaghini [1,*]

1 Department of Surgical Biotechnology, Division of Surgery and Interventional Science, University College London, London NW3 2QG, UK; andrea.balukova.16@ucl.ac.uk (A.B.); kalliopi.bokea.19@ucl.ac.uk (K.B.)
2 Department of Oncology, UCL Cancer Institute, University College London, London WC1E 6DD, UK; p.barber@ucl.ac.uk
3 Comprehensive Cancer Centre, School of Cancer and Pharmaceutical Sciences, King's College London, London SE1 9RT, UK; simon.ameer-beg@kcl.ac.uk
* Correspondence: a.macrobert@ucl.ac.uk (A.J.M.); elnaz.yaghini@ucl.ac.uk (E.Y.)

Supporting Information

In the 3D compressed collagen model, constructs were cultivated for 1 week and then treated with 1 μM $TPPS_{2a}$, for 24 hours. Tme-lapse confocal imaging was used to assess the changes in PS fluorescence intensity over time. An image was captured every minute for a duration of 5 minutes using a 405 nm laser for excitation at 100% intensity. For the time-lapse image analysis, MATLAB was used to generate a false colour intensity map. The $TPPS_{2a}$ fluorescence was notably dissipated after 3 min of light exposure, with further diminution evident after 5 minutes. (Figure S1).

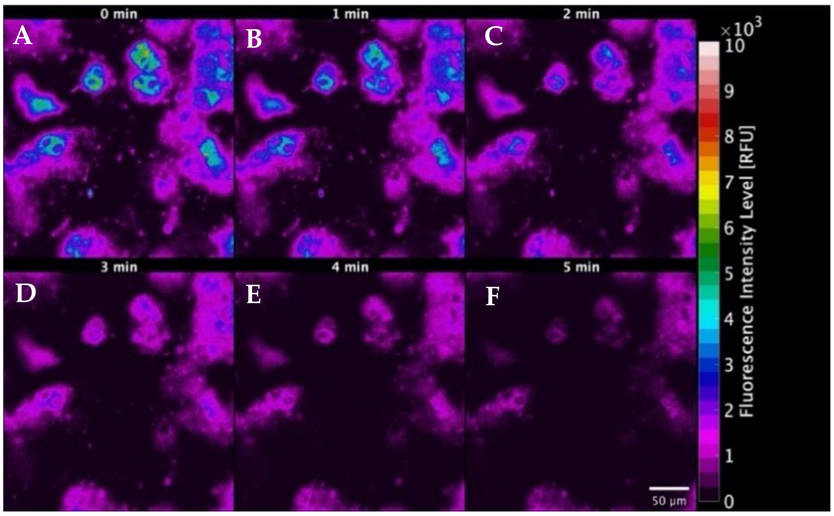

Figure S1. Confocal images of HEY 3D compressed spheroid construct. Constructs were treated with 1 μM porphyrin $TPPS_{2a}$, which was excited using the 405 nm laser. The montage presents a colour intensity map showing the decrease in $TPPS_{2a}$ fluorescence every 1 minute as laser exposure time increases. The scale bar presented in image F is 50 μm and is representative for all images.

To show the spheroidal structure of the 3D compressed collagen constructs, z-stack images were obtained on 7-day grown constructs, which were fixed and stained with DAPI (Figure S2).

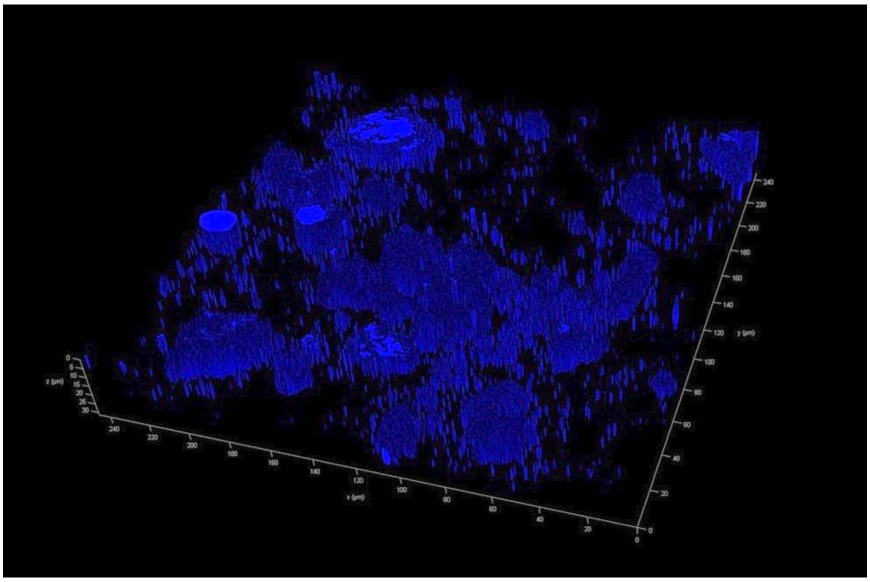

Figure S2. A z-stack 3D image of a 3D compressed collagen construct stained only with DAPI showing the spheroids. The x and y axis presented are 240 microns, and the z axis is 30 microns.

For the investigation of Dihydroethidium (DHE) as a detector of superoxide anion, both 2D and 3D models were co-treated with the porphyrin $TPPS_{2a}$ and DHE. For the 2D model, cells were incubated with 2 μM $TPPS_{2a}$ for 22 hours after which the DHE was added for 2 hours at 10 μM. In 2D model, an insignificant 15% ± 3% increase in DE fluorescence was detected in cells following 5 minutes light illumination in co-treated sample (Figure S3 A, B).

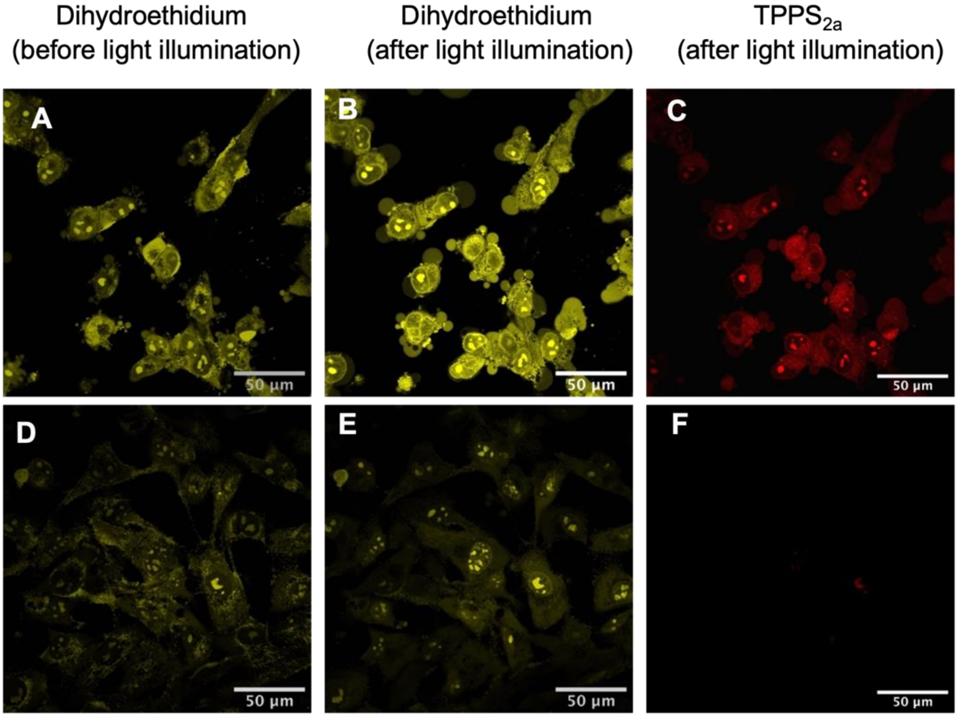

Figure S3. Confocal images of HEY 2D monolayer culture. (A, B, C): Cells were incubated with 2 μM $TPPS_{2a}$ for 22 hours and 10 μM DHE for a further 2 hours. (D, E, F): Cells were incubated only with 10 μM DHE for 2 hours without $TPPS_{2a}$. Illumination was carried out at 405 nm. The scale bar presented in each image is 50 μm.
